# Improving the safety of nasogastric tube insertion by the “SORT maneuver” during the novel coronavirus pandemic (COVID-19)

**DOI:** 10.1186/s13037-020-00280-4

**Published:** 2021-01-06

**Authors:** Mahdi Najafi

**Affiliations:** 1 Schulich School of Medicine & Dentistry, Western University, Room M402, Medical Sciences Building, 1151 Richmond St, N6A 3K7 London, Ontario Canada; 2 Department of Anesthesiology, Tehran Heart Center, Tehran University of Medical Sciences, Tehran, Iran

**Keywords:** Nasogastric tube, Insertion, Complications, Outbreak, COVID-19, Infection prevention

The "SORT maneuver" (sniffing position, NGT orientation, contralateral rotation, and twisting movement) was introduced in 2016 to ease nasogastric intubation for unconscious patients in operating theatre and critical care setting [1]. This technique is aimed at a safe practice without using equipment. Safety has never been more important than the current outbreak of SARS-CoV-2 infection. SORT maneuver can be of benefit for both the healthcare workers and the patients who are struggling with coronavirus during this pandemic.

From the practitioner’s point of view, any procedure which is done in close contact with an infected patient’s airway carries a higher risk of respiratory infection transmission which may not get totally eliminated after tracheal intubation [2, 3]. With regards to NGT intubation, this is supported by updated guidelines that consider placement of any enteral access device as an aerosol-generating procedure [4]. Moreover, NGT insertion may expose healthcare workers to infectious saliva of patients with coronavirus [5]. Feasibility of NGT insertion and the number of attempts for intubation matter as well, when it comes to a safe bedside practice. The first clinical trial reporting successful employment of SORT maneuver for NGT insertion in unconscious patients admitted to intensive care unit was just recently published with promising results [6]. This study showed that ease of insertion was considerably greater and success rate was much higher for SORT maneuver than the other conventional technique of neck flexion and lateral pressure. The observation that SORT was “easy to learn by unskilled providers” is an asset in a crisis like COVID-19 outbreak [7]. Also, compared to this technique which is based on anatomical approach, NGT insertion using laryngoscope and/or Magill forceps can even increase the risk substantially as it is more invasive and requires closer proximity to the airway [8]. Although trachea is intubated, security of airway is not guaranteed at least in circumstances such as ventilation at high inspiratory pressure, sputum induction, and manual ventilation especially with undersized or uncuffed tracheal and tracheostomy tubes [9].

On the other hand, the management of the patients’ coexisting problems such as cardiovascular disease and hemodynamic instability is a challenge during NGT intubation. Pharyngeal manipulation during NGT insertion is a potential treat for cardiac patients by increasing demand especially in hypertensive patients with uncontrolled blood pressure. These patients are the most vulnerable group to higher incidence of severe illness and worse outcome in COVID-19 as well [10, 11]. Smooth process of NGT insertion without equipment using SORT maneuver, is capable of preventing from brisk hemodynamic response and its adverse effects [12]. If this is also of benefit for infected patients with coronavirus, is a question to be addressed. Using paralyzing drugs whenever is indicated, mitigates patient’s reactions and probably can reduce the risk [3]. However, unlike the operating theatre, we seldom use these agents in intensive care unit.

NGT tube insertion in intubated patient is a quite common procedure in operation theatre and intensive care settings. In patients with COVID-19 infection, SORT maneuver may protect both practitioners and patients from further avoidable hazards. I would encourage my colleagues to verify these proposals in their daily practice and by further investigations through clinical trials.

## Data Availability

Data sharing does not apply to this article as no datasets were generated or analyzed during the current study.

## Abbreviations

COVID-19: Coronavirus disease 2019
NGT: Nasogastric tube
